# The inotropic and arrhythmogenic effects of acutely increased late I_Na_ are associated with elevated ROS but not oxidation of PKARIα

**DOI:** 10.3389/fcvm.2024.1379930

**Published:** 2024-07-15

**Authors:** Theresa Gissibl, Laura Stengel, Daniel Tarnowski, Lars S. Maier, Stefan Wagner, Anna-Lena Feder, Can Martin Sag

**Affiliations:** Department of Internal Medicine II/Cardiology, University Medical Center Regensburg, Regensburg, Germany

**Keywords:** PKARIα, excitation contraction coupling (ECC), CaMKII, oxidative stress, L-type Ca^2+^ current (I_Ca_)

## Abstract

**Background:**

Acute stimulation of the late sodium current (I_NaL_) as pharmacologically induced by Anemonia toxin II (ATX-II) results in Na^+^-dependent Ca^2+^ overload and enhanced formation of reactive oxygen species (ROS). This is accompanied by an acute increase in the amplitude of the systolic Ca^2+^ transient. Ca^2+^ transient amplitude is determined by L-type Ca^2+^-mediated transsarcolemmal Ca^2+^ influx (I_Ca_) into the cytosol and by systolic Ca^2+^ release from the sarcoplasmic reticulum (SR). Type-1 protein kinase A (PKARIα) becomes activated upon increased ROS and is capable of stimulating I_Ca_, thereby sustaining the amplitude of the systolic Ca^2+^ transient upon oxidative stress.

**Objectives:**

We aimed to investigate whether the increase of the systolic Ca^2+^ transient as acutely induced by I_NaL_ (by ATX-II) may involve stimulation of I_Ca_ through oxidized PKARIα.

**Methods:**

We used a transgenic mouse model in which PKARIα was made resistant to oxidative activation by homozygous knock-in replacement of redox-sensitive Cysteine 17 with Serine within the regulatory subunits of PKARIα (KI). ATX-II (at 1 nmol/L) was used to acutely enhance I_NaL_ in freshly isolated ventricular myocytes from KI and wild-type (WT) control mice. Epifluorescence and confocal imaging were used to assess intracellular Ca^2+^ handling and ROS formation. A ruptured-patch whole-cell voltage-clamp was used to measure I_NaL_ and I_Ca_. The impact of acutely enhanced I_NaL_ on RIα dimer formation and PKA target structures was studied using Western blot analysis.

**Results:**

ATX-II increased I_NaL_ to a similar extent in KI and WT cells, which was associated with significant cytosolic and mitochondrial ROS formation in both genotypes. Acutely activated Ca^2+^ handling in terms of increased Ca^2+^ transient amplitudes and elevated SR Ca^2+^ load was equally present in KI and WT cells. Likewise, cellular arrhythmias as approximated by non-triggered Ca^2+^ elevations during Ca^2+^ transient decay and by diastolic SR Ca^2+^-spark frequency occurred in a comparable manner in both genotypes. Most importantly and in contrast to our initial hypothesis, ATX-II did not alter the magnitude or inactivation kinetics of I_Ca_ in neither WT nor KI cells and did not result in PKARIα dimerization (i.e., oxidation) despite a clear prooxidant intracellular environment.

**Conclusions:**

The inotropic and arrhythmogenic effects of acutely increased I_NaL_ are associated with elevated ROS, but do not involve oxidation of PKARIα.

## Introduction

Impaired intracellular sodium (Na^+^) and calcium (Ca^2+^) handling is a key feature of heart failure (HF) contributing to both systolic and diastolic dysfunctions as well as to cardiac arrhythmias (1). An important component of altered Na^+^ handling in HF is the late sodium current I_NaL_. Most Na^+^ channels open transiently during the upstroke of the action potential and then close rapidly, forming the peak flow required for action potential generation and conduction. A few Na^+^ channels, however, fail to inactivate and thus enable the so-called late sodium current (I_NaL_) to persist over hundreds of milliseconds during the plateau phase of the action potential (2). In HF, enhanced I_NaL_ (3) contributes to increased Na^+^ influx into the cell, resulting in prolongation of action potential duration and arrhythmogenesis (4). In addition, intracellular Na^+^ is closely linked to Ca^2+^ handling via the Na^+^-Ca^2+^ exchanger (NCX). Increased intracellular Na^+^ concentration [Na^+^]_i_ leads to a reduced electrochemical gradient for Ca^2+^ extrusion. Instead, the NCX operates in a “reverse mode” through which Ca^2+^ is transported into the cell and Na^+^ out of it (5). The subsequent excessive Ca^2+^ influx into the cell contributes to diastolic Ca^2+^ overload in chronic HF (6), as well as to arrhythmias (2) and contractile dysfunction (7). On the cellular level, I_NaL_-related Na^+^-dependent Ca^2+^ overload results in pathological overactivation of protein kinases including Ca^2+^/Calmodulin-dependent protein kinase II (CaMKII) (8) and cAMP-dependent protein kinase A (PKA) (9), which is accompanied by proarrhythmic diastolic Ca^2+^ loss from the sarcoplasmic reticulum (SR), and mitochondrial reactive oxygen species (ROS) formation (10, 11).

Increased ROS formation results in oxidative stress when ROS levels exceed the antioxidative capacity of myocytes, which represents a well-established pathomechanism of various cardiac diseases including HF (12, 13). ROS serve as signaling molecules under physiological and disease-related conditions and directly influence the function of various channels and transporters in the heart through oxidation, such as those of the ryanodine receptor (RyR2) or SERCA2a (14). Besides, modulation of kinase function through oxidation has been reported for, e.g., CaMKII (15) and type I PKA (16), both of which influence the function of target structures. In that regard, Viatchenko-Karpinski et al. have already demonstrated that increased I_NaL_ with subsequent cytosolic Na^+^ and Ca^2+^ overload leads to oxidation of CaMKII resulting in dysregulation of Ca^2+^ handling (17). Moreover, Eiringhaus et al. reported that PKA activity was also increased upon elevated I_NaL_ as induced experimentally by Anemonia sulcata toxin II (ATX-II), which was associated with an acute positive inotropic effect that could be blocked pharmacologically using the PKA-inhibitor H89 (9). However, the authors did not assess whether increased PKA activity following ATX-II is solely due to a cAMP-dependent mechanism or may also involve oxidative activation.

PKA is the main effector of beta-adrenergic stimulation in the heart and gets activated in a cAMP-dependent manner. It is composed of two regulatory (R) and two catalytic (C) subunits. Depending on the R subunit, RI or RII, the enzyme is defined as type I or type II PKA (18). Brennan et al. were the first to demonstrate a cAMP-independent activation of type I PKA upon elevated ROS induced by H_2_O_2_ exposure. The subunit RIα contains redox-sensitive Cysteine residues forming interprotein disulfide bonds upon oxidation resulting in dimerization of the regulatory subunits and subsequent kinase activation independent of cAMP (16). The pathophysiological significance of oxidative activation of PKARIα in the complex interaction of the various redox-sensitive mechanisms in cardiomyocytes is still a subject of intense research. The clinical relevance of PKARIα has already been shown for tumor-, vascular endothelial growth factor (VEGF)-, and ischemia-induced angiogenesis (19) as well as for platelet-derived growth factor (PDGF) signaling pathways in renal mesangial cells (20). Recent research has focused on elucidating the role of redox-dependent activation of PKARIα in cardiomyocytes. In that regard, Trum et al. found that redox-activated PKARIα inhibits potassium channels and thereby contributes to early after depolarizations suggesting a harmful role for PKARIα in cardiac physiology (21). By contrast, Simon et al. showed that oxidative activation of PKARIα exerts a protective effect in the context of ischemia–reperfusion damage, presumably by inhibiting excessive lysosomal-mediated Ca^2+^ release (22). In line with this, we have recently found protective effects of oxidized PKARIα with respect to cardiocellular and ventricular contractile functions in the context of acute and chronic oxidative stress through enhanced L-type Ca^2+^ channel (LTCC) mediated Ca^2+^ influx (I_Ca_) into ventricular myocytes that results in partially maintained systolic Ca^2+^ transients despite oxidative stress (23).

However, it is not clear if and to what extent ROS formation may lead to oxidation of PKARIα upon acute ATX-II-mediated Na^+^/Ca^2+^ overload and whether oxidized PKARIα is required for the inotropic and arrhythmogenic effects of ATX-II as reported earlier (9). Therefore, we made use of a genetically altered redox-dead mouse model with homozygous knock-in replacement of a redox-sensitive Cysteine 17 with Serine within the regulatory subunits of PKARIα (KI) (19, 22, 23) to test whether acutely induced I_NaL_ [by (ATX-II)] may oxidize PKARIα. We acutely exposed KI and wild-type (WT) control ventricular myocytes to ATX-II and comprehensively studied intracellular Ca^2+^ handling in an *in vitro* model.

## Material and methods

### Animals

Ventricular myocytes were isolated from a redox-dead transgenic mouse model in which a redox-sensitive Cysteine was made resistant to oxidative activation by knock-in replacement with Serine (PKARIαCys17Ser knock-in mouse model, KI). The point mutation of Cys17Ser was introduced into exon 1 of the Prkar1a gene as described previously (19). Ventricular myocytes from wild-type littermates were used as control. All animal procedures were performed in accordance with the Guide for the Care and Use of Laboratory Animals and approved by the Institutional Animal Care and Use Committee.

### Cardiomyocyte isolation

The isolation of ventricular cardiomyocytes was performed as previously reported (23) using adult (12–15 weeks) mice of both sexes in equal shares. Hearts were mounted on a Langendorff perfusion apparatus and were retrogradely perfused for 7–9 min, starting with a calcium-free solution containing (in mmol/L): NaCl 113, KCl 4.7, KH_2_PO_4_ 0.6, Na_2_HPO_4_ 0.6, MgSO_4_ 1.2, phenol-red 0.032, NaHCO_3_ 12, KHCO_3_ 10, HEPES 10, taurine 30, BDM (2,3 butanedione monoxime) 10, and glucose 5.5 (at 37°C, pH 7.4). After 4 min, trypsin 0.6% (Thermo Fisher Scientific Inc. of Waltham, MA, USA), 7.5 mg/ml Liberase™ (Roche Diagnostics, Mannheim, Germany), and 0.125 mmol/L CaCl_2_ were added and the hearts were perfused until they became flaccid. After enzymatic digestion, the ventricular myocardium was dissected from the atrium and mechanical dissociation of the heart was performed in a solution containing 10% bovine calf serum (BCS; Sigma-Aldrich, St. Louis, MO, USA). For immediate measurements, a gradual Ca^2+^ reintroduction was performed from 0.1 to 0.8 mmol/L. The isolated cardiomyocytes were plated on laminin-coated glass coverslips. Cardiomyocytes were allowed to settle for 15 min at room temperature to allow cell adhesion for the following experiments.

### Chemicals and experimental solutions

ATX-II, which was used to increase I_NaL_, was purchased from Abcam (ab141870, Cambridge, Great Britain). The sources of the fluorescent dyes and antibodies are given in the following corresponding sections. If not indicated otherwise, Standard Tyrode's solution was used as the experimental solution, containing (in mmol/L) NaCl 140, KCl 4, HEPES 5, MgCl_2_ 1, glucose 10, and CaCl_2_ 1. The pH value was adjusted to 7.40 at 37°C (referred to as “normal Tyrode's solution”, NT).

### Patch clamp experiments

Ruptured-patch whole-cell voltage-clamp was used to measure I_NaL_ and I_Ca_ as previously reported (23–25). The myocytes were mounted on the stage of a microscope (Nikon TE2000-U). They were incubated for 15 min with the respective bath solution before measurements were undertaken. Notably, I_NaL_ and I_Ca_ measurements were performed within the same time limits as the Ca^2+^ imaging experiments (i.e., following at least 10 min of ATX-II exposure). The experimental groups were additionally treated with 1 nmol/L ATX-II. Cardiomyocytes typically achieved a seal greater than 1 GΩ and a resistance <10 MΩ after rupture. For I_NaL_ measurements, the microelectrodes (2–3 MΩ) were filled with (in mmol/L) the following: CsCl 95, Cs-glutamate 40, NaCl 10, MgCl_2_ 0.92, Mg-ATP 5, Li-GTP 0.3, HEPES 5, niflumic acid 0.03, nifedipine 0.02, strophanthidin 0.004, EGTA 1, and CaCl_2_ 0.36 [free (Ca^2+^)_i_, 100 nmol/L, pH 7.2, CsOH]. The bath solution contained (in mmol/L) the following: NaCl 135, tetramethylammonium chloride 5, CsCl 4, MgCl_2_ 2, glucose 10, and HEPES 10 (pH 7.4, CsOH). To measure I_NaL_, the cardiomyocytes were held at −120 mV, and I_NaL_ was elicited using a train of pulses to −35 mV (1,000 ms duration, 10 pulses, basic cycle length (BCL) 2 s). Recordings were initiated 3–4 min after rupture and were performed at room temperature. The measured current was integrated (between 100 and 500 ms) and normalized to membrane capacitance. For I_Ca_ measurements, the microelectrodes (2–3 MΩ) were filled with (in mmol/L) the following: CsCl 86, Cs-glutamate 40, MgCl_2_ 0.92, Mg-ATP 5, Li-GTP 0.3, HEPES 10, EGTA 5, and CaCl_2_ 1.8 [free (Ca^2+^)_i_ 100 nmol/L, pH 7.2, CsOH]. The bath solution contained (in mmol/L) the following: NaCl 140, CsCl 4, MgCl_2_ 1, glucose 10, HEPES 10, and CaCl_2_ 1 (pH 7.4, CsOH). The signals were filtered with 2.9 and 10 kHz Bessel filters and recorded with an EPC10 amplifier (HEKA Elektronik). The recordings were started 2–3 min after rupture and conducted at room temperature. I_Ca_ analysis was performed using LabChart 10 (ADInstruments) and determined by subtracting the steady-state current from the peak I_Ca_ current. Subsequently, the amplitude was normalized to cell capacitance. For analysis of I_Ca_ inactivation kinetics, time constants *τ*_slow_ (tau slow) and *τ*_fast_ (tau fast) were calculated by biexponential fitting of I_Ca_ currents using ClampFit.

### Assessment of intracellular Ca^2+^ by epifluorescence microscopy

For the assessment of cytosolic Ca^2+^, isolated cardiomyocytes were loaded with 10 µmol/L Fura-2-AM (Thermo Fisher Scientific Inc. of Waltham, MA, USA) in the presence of 0.02% (w/v) pluronic acid (Molecular Probes, Eugene, OR, USA) for 15 min at room temperature in darkness (23, 25). Afterward, the cells were incubated for another 15 min in NT to ensure complete deesterification of intracellular Fura-2. After deesterification, Fura-2-loaded cardiomyocytes were mounted on the stage of a Nikon Eclipse TE200-U inverted epifluorescence microscope. Fura-2 was alternately excited at 340 ± 5 and 380 ± 5 nm (250 pairs per second, HyperSwitch system, IonOptix Corp., Westwood, MA, USA) and emitted fluorescence was collected at 510 ± 20 nm. Excitation light was provided by a 75 W xenon arc lamp (Ushio, Japan). During measurements, the cardiomyocytes were continuously transilluminated with red light (>650 nm), stimulated at a frequency of 0.5 Hz, and superfused with experimental solution (with vehicle control or ATX-II, respectively) at a flow rate of 50 ml/h at 37°C. To estimate SR Ca^2+^ content, Ca^2+^ transient amplitude was determined after rapid caffeine application (10 mmol/L, Sigma-Aldrich) during field stimulation pause. After subtraction of background fluorescence, cytosolic Ca^2+^ levels were assessed as the 340/380 nm fluorescence ratio (F_340_/F_380_). Simultaneously with Ca^2+^ measurements, sarcomere length and fractional shortening of the myocytes were recorded using a sarcomere length detection system (MyoCam, IonOptix Corporation, Westwood, MA, USA). Contractions and Ca^2+^ transients were averaged over about 10 beats for the analysis of all parameters. Recorded data were analyzed with the software IONWizard (IonOptix Corp.).

### Assessment of Ca^2+^ spark frequency and ROS formation by confocal microscopy

**Figure 1:**
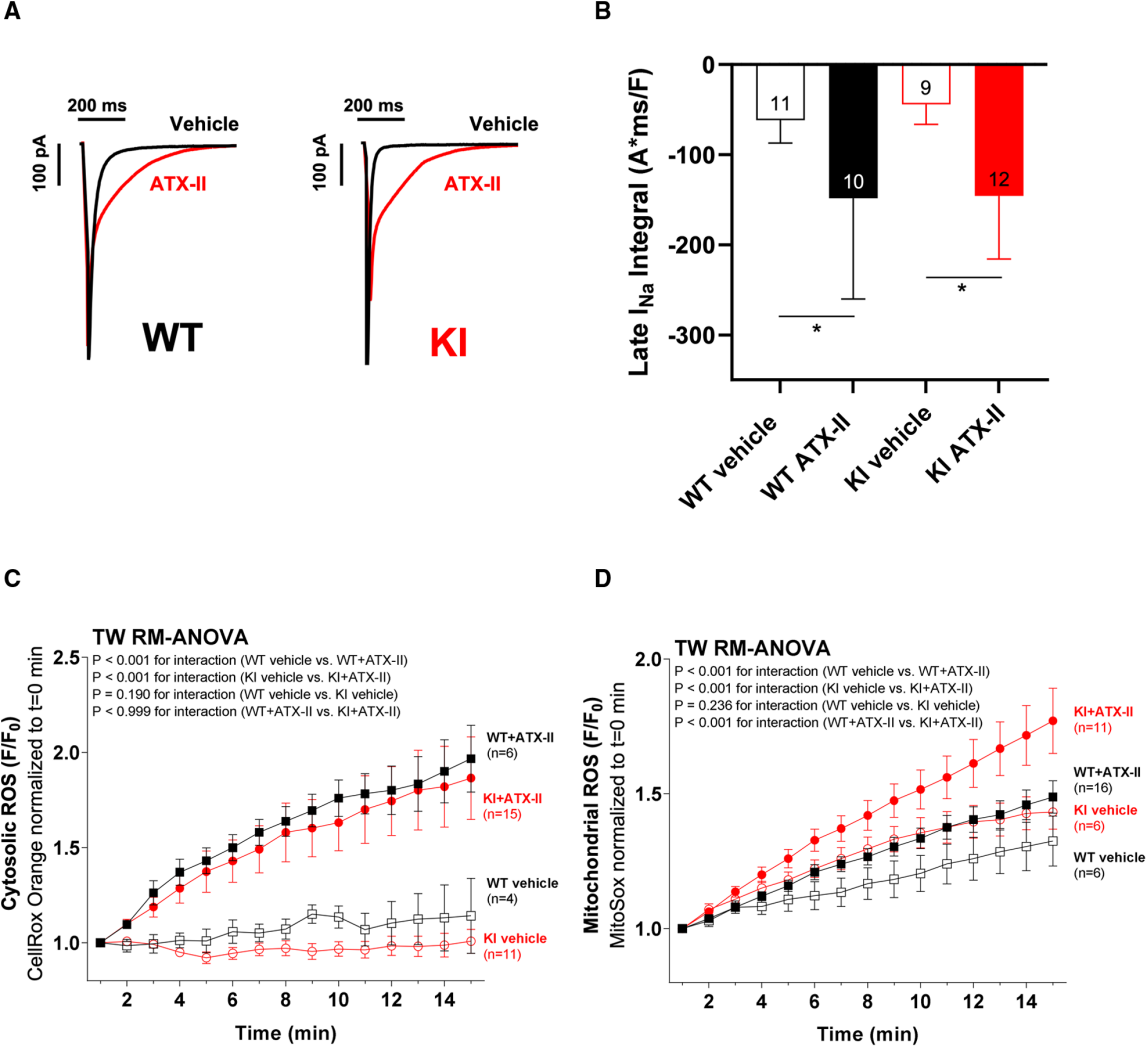
ATX-II increases I_NaL_ in WT and KI myocytes and leads to elevated cytosolic and mitochondrial ROS production. Original recordings of I_NaL_ (**A**) and mean data of the I_NaL_ integral (**B**) demonstrate an approximately threefold increase of I_NaL_ upon treatment with ATX-II in both WT and KI murine ventricular myocytes. Mean data normalized to fluorescence at *t* = 0 min of isolated ventricular WT and KI myocytes loaded with ROS sensor CellRox Orange (**C**) and MitoSox Red (**D**) show increased cytosolic (**C**) and mitochondrial (**D**) ROS formation in cells upon perfusion with ATX-II in comparison with vehicle-treated control myocytes. * indicates significance between groups using the Holm–Sidak *post-hoc* test.

Ca^2+^ spark frequency was analyzed using a laser scanning confocal microscope (Zeiss LSM 700 Pascal; Göttingen, Germany). Isolated cardiomyocytes were loaded with 10 µmol/L Fluo-4-AM (Thermo Fisher Scientific Inc. of Waltham, MA, USA) in the presence of 0.02% (w/v) pluronic acid (Molecular Probes, Eugene, OR, USA) for 15 min at room temperature in darkness (23). Afterward, the solution was replaced by Tyrode's solution. The cardiomyocytes were left to incubate for further 15 min for complete deesterification until the Fluo-4-loaded cardiomyocytes were placed on the stage of a laser scanning confocal microscope. Measurements were conducted in the line-scan mode (10,000 lines per scan). The cardiomyocytes were excited at 488 nm using an argon laser and emission was collected at 505–530 nm through a long-pass emission filter under continuous superfusion with experimental solution (with vehicle or 1 nmol/L ATX-II) and field stimulation at 0.5 Hz at 37°C. Ca^2+^ sparks were detected and quantified using SparkMaster with manual detection of sparks. Ca^2+^ spark frequency (CaSpF) was calculated and normalized to scanning interval and cell width. Only cells that showed stimulated Ca^2+^ transients were included in the evaluation to avoid misinterpretation by an elevated Ca^2+^ spark frequency in non-viable cells. To assess mitochondrial ROS formation, isolated ventricular cardiomyocytes were loaded with 5 µmol/L MitoSox Red (Thermo Fisher Scientific Inc. of Waltham, MA, USA) in presence of 0.02% (w/v) pluronic acid (Molecular Probes, Eugene, OR, USA) for 15 min at 37°C. The cardiomyocytes were excited at 488 nm using an argon laser and fluorescence emission was collected at 610 nm through a long-pass emission filter. Once every minute, frame scans were acquired and MitoSox Red fluorescence emission was normalized to the initial fluorescence (expressed as F/F_0_). Likewise, cytosolic ROS formation was assessed by excitation of CellRox Orange-loaded cardiomyocytes at 555 nm while fluorescence emission was collected at 570 nm (Thermo Fisher Scientific Inc. of Waltham, MA, USA, incubation for 30 min with 5 µmol/L at 37°C). Please note that mitochondrial ROS formation as depicted in Figure 1D was assessed in independent sets of experiments in WT vs. KI myocytes with higher initial MitoSox fluorescence in KI cells at baseline (1,665 ± 188 a.u.) as compared with WT (1,004 ± 52 a.u.).

### Detection of protein expression and phosphorylation levels by Western blotting

For Western blot analysis, the cardiomyocytes were treated with vehicle or ATX-II (1 nmol/L) and incubated for 15 min at room temperature. The harvested cardiomyocytes were frozen immediately. The proteins were denatured for 5 min at 95°C under non-reducing conditions for PKARIα dimer and PKARIα monomer. To assess pSer2809 RyR2, pSer2814 RyR2, RyR2, pSer16 phospholamban (PLB), and PLB, the proteins were denatured for 30 min at 37°C in presence of 10% β-mercaptoethanol. They were denatured for 5 min at 95°C in presence of 10% β-mercaptoethanol for assessment of pThr287 CaMKII and CaMKIIδ expression. Following denaturation, the proteins were separated on 5% (RyR2, pSer2809 RyR2, pSer2814 RyR2), 8% (PKARIα dimer, PKARIα monomer, pThr287 CaMKII, and CaMKIIδ), or 12.5% (PLB, pSer16 PLB) SDS-polyacrylamide gels, transferred to a nitrocellulose membrane and incubated with the following primary antibodies: rabbit polyclonal anti-pThr287 CaMKII (1:1,000, PhosphoSolutions, Aurora, CO, USA), rabbit polyclonal anti-CaMKIIδ (1:10.000, Thermo Fisher Scientific Inc. of Waltham, MA, USA), mouse monoclonal anti-PKARIα (1:1,000, BD Biosciences, Heidelberg, Germany), rabbit polyclonal anti-RyR2 antibody (1:10.000, Sigma-Aldrich, St. Louis, MO, USA), rabbit polyclonal anti-pSer2809 RyR2 antibody (1: 1,000, Badrilla, Leeds, United Kingdom), rabbit polyclonal anti-pSer2814 RyR2 antibody (1:1,000, Badrilla, Leeds, United Kingdom), mouse monoclonal anti-PLB (1:10.000, Thermo Fisher Scientific Inc. of Waltham, MA, USA), rabbit polyclonal anti-pSer16 PLB (1:500, Badrilla, Leeds, United Kingdom), and mouse monoclonal anti-GAPDH (1:10.000, Sigma-Aldrich, St. Louis, MO, USA) at 4°C overnight. Secondary antibodies were horseradish peroxidase (HRP)-conjugated sheep anti-mouse and donkey anti-rabbit IgG (1:10.000, GE Healthcare, Chicago, Illinois, USA) and incubated for 1 h at room temperature. For chemiluminescent detection, Immobilon™ Western Chemiluminescent HRP Substrate (Millipore) was used. The values were normalized to GAPDH and WT vehicle values later.

### Statistical analysis and data visualization

Data are presented as means ± standard error of the mean (SEM). Statistical analyses were performed using unpaired Student's *t*-test, one-way ANOVA, and two-way ANOVA for repeated measurements (RM) with the Holm–Sidak *post-hoc* test and Mantel–Cox test for survival. Values of *p* < 0.05 were considered as statistically significant. The graphs were created using Sigma Plot 12 and Graph Pad Prism 8.0.1.

## Results

### ATX-II increases I_NaL_ in WT and KI myocytes and leads to elevated cytosolic and mitochondrial ROS production

In a first step, we wanted to exclude the possibility that the knock-in replacement of Cys17 with Serine in the regulatory subunit of PKARIα might lead to a different responsiveness of I_NaL_ to ATX-II in KI cells. Therefore, we exposed isolated ventricular cardiomyocytes of both genotypes to 1 nmol/L ATX-II. As depicted in the original measurements in Figure 1A, ATX-II led to an approximately threefold increase of I_NaL_ in both WT and KI cells (mean values of the I_NaL_ integral are shown in Figure 1B). As previously reported by Viatchenko-Karpinski et al. (17), this increase in I_NaL_ was associated with significant cytosolic (Figure 1C) and mitochondrial ROS production (Figure 1D) in both genotypes in our model as well.

### ATX-II activates Ca^2+^ handling in WT and KI cardiomyocytes independent of oxidized PKARIα

**Figure 2:**
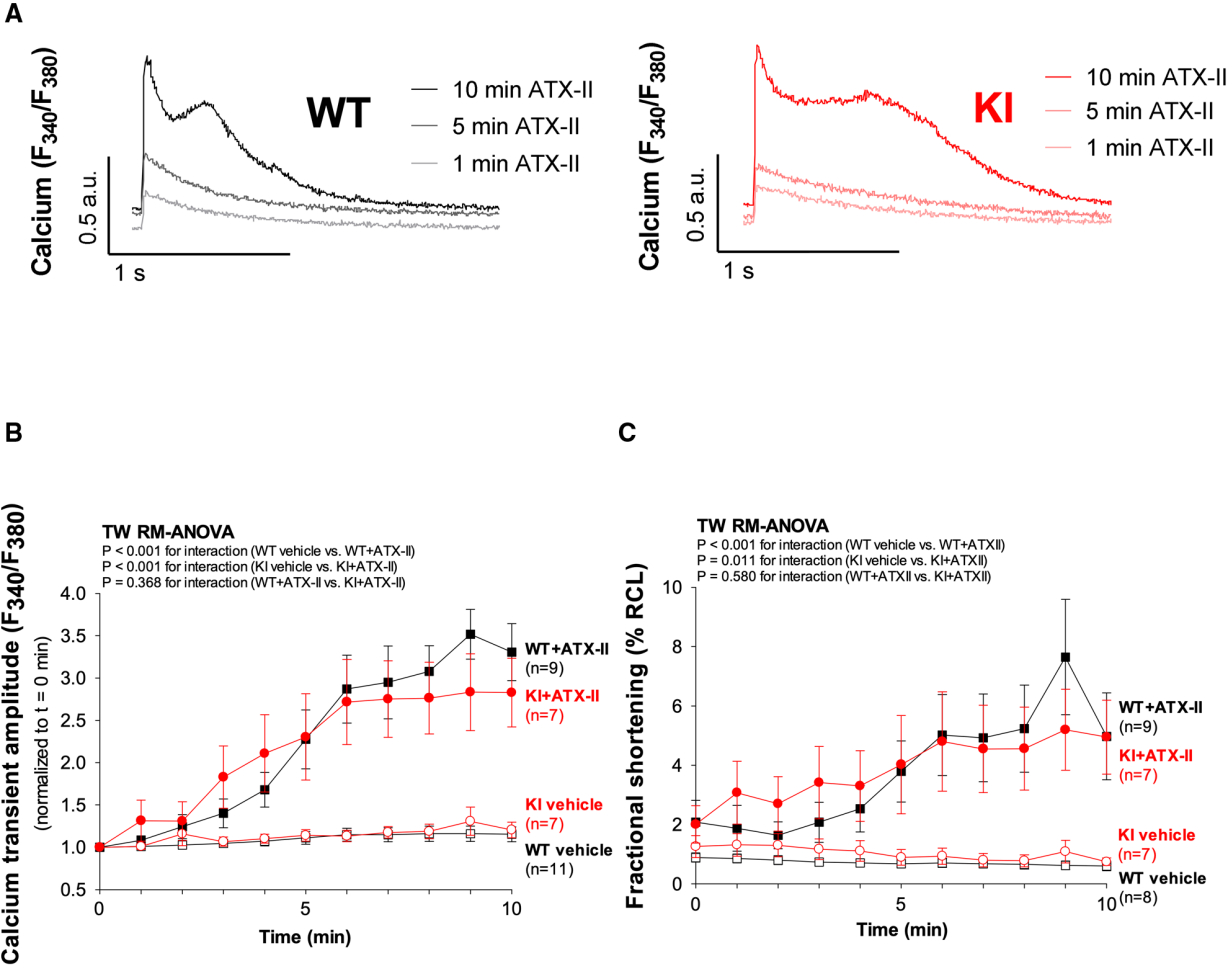
ATX-II activates Ca^2+^ handling in WT and KI cardiomyocytes independent of oxidized PKARIα. (**A**) Original traces of intracellular Ca^2+^ transients measured in Fura-2 AM-loaded ventricular myocytes from WT and PKARIα KI mice at baseline, after 5 min and after 10 min exposure to ATX-II. (**B**) Mean data for Ca^2+^-transient amplitudes normalized to *t* = 0 min during treatment with ATX-II for 10 min show a significant approximately threefold increase in both WT and KI ventricular myocytes to a similar extent. (**C**) The positive inotropic effect of ATX-II (1 nmol/L) treatment is also represented in the mean data for fractional shortening of myocytes [expressed as % resting cell length (%RCL)].

**Figure 3:**
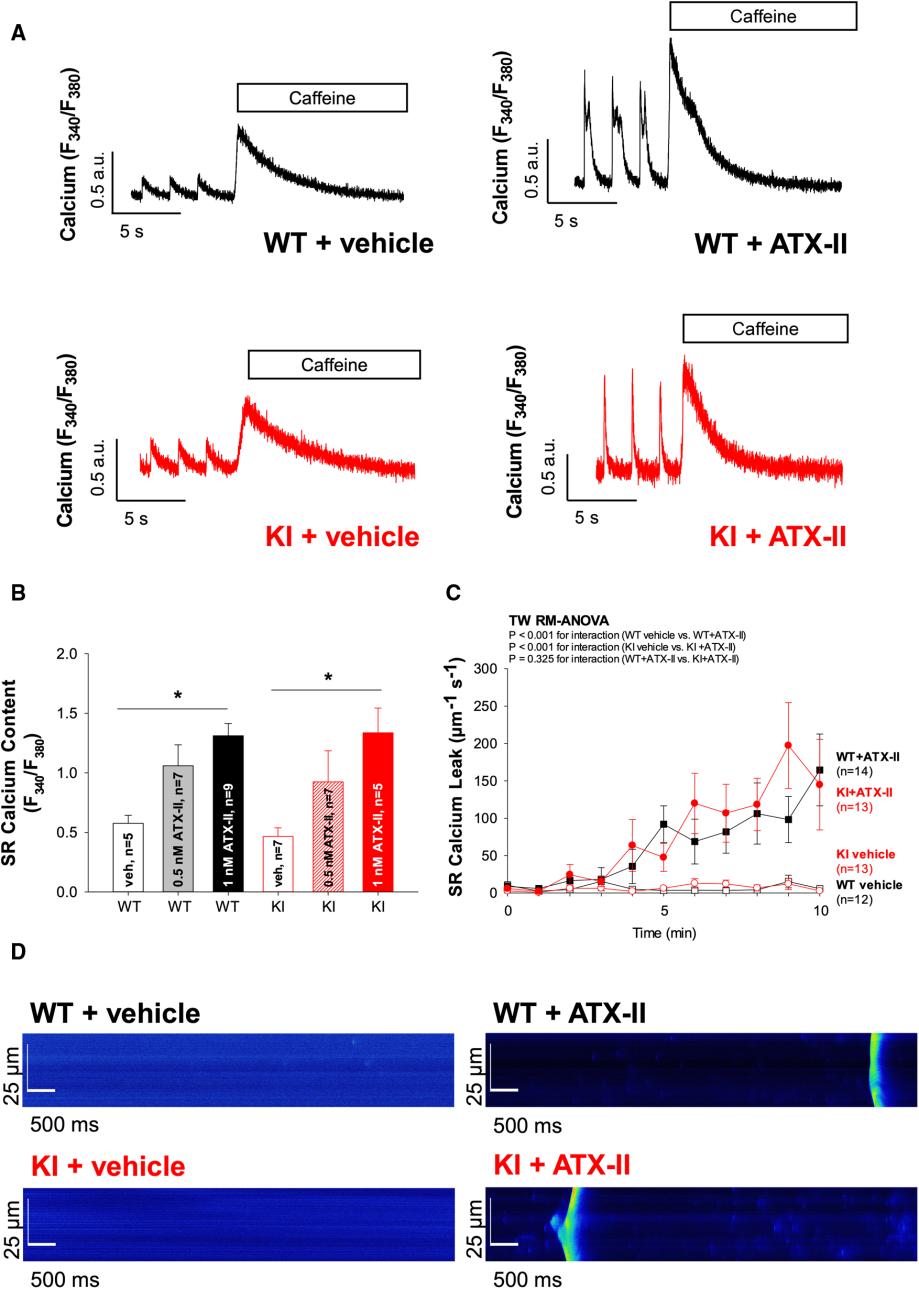
Exposure to ATX-II leads to an increased SR Ca^2+^ loading associated with increased diastolic SR Ca^2+^ spark frequency. (**A**) Original traces of caffeine-induced (10 mmol/L) Ca^2+^ transients to assess SR Ca^2+^ content upon exposure to vehicle vs. ATX-II (1 nmol/L) in WT and KI ventricular myocytes. (**B**) Mean data show that SR Ca^2+^ content is significantly increased upon treatment with ATX-II (1 nmol/L) in a dose-dependent manner in isolated ventricular myocytes of both WT and KI mice without a significant difference between genotypes. (**C**) Mean data for diastolic Ca^2+^ spark frequency illustrate an increased SR Ca^2+^ leak in WT and in KI cardiomyocytes upon treatment with ATX-II (1 nmol/L). (**D**) Original confocal line-scan images of isolated ventricular myocytes of WT and KI mice loaded with Fluo-4 at baseline and after 10 min of perfusion with ATX-II (1 nmol/L). * indicates significance between groups using the Holm–Sidak *post-hoc* test.

In a next step, we aimed to investigate whether oxidatively activated PKARIα is required for the ATX-II-dependent and PKA-mediated activation of intracellular Ca^2+^ handling that has been previously described by Eiringhaus et al. (9). As shown in Figures 2A,B, ATX-II caused a fairly immediate and time-dependent increase in the amplitude of the systolic Ca^2+^ transient that reached its maximum about 6–10 min after infusion of ATX-II. However, this pronounced, approximately threefold increase in systolic Ca^2+^ transient amplitude was largely comparable between WT and KI cells, which indicates that oxidatively activated PKARIα is functionally not required here (*p* = 0.368 for interaction, see Figure 2B). In line with this, a positive inotropic effect in terms of time-dependently increased fractional myocytes shortening was comparably observed in both WT and KI cells (Figure 2C). Further, rapid caffeine application was used to assess SR Ca^2+^ content in WT and KI myocytes (Figures 3A,B). Original traces in Figure 3A show that SR Ca^2+^ content was significantly increased by ATX-II treatment in both WT and KI myocytes without major differences between genotypes. The mean values in Figure 3B illustrate dose-dependency of SR Ca^2+^ load following ATX-II treatment in both genotypes without significant differences between WT and KI cells (*p* = 0.873 for interaction). The ATX-II-dependent rise in SR Ca^2+^ content was further associated with a time-dependent increase in the frequency of diastolic Ca^2+^ spark events as a measure of diastolic Ca^2+^ leakage from the SR (see Figure 3C, original confocal line-scans are shown in Figure 3D). Equivalent to Ca^2+^ transient amplitudes and SR Ca^2+^ content, this ATX-II-dependent increase in Ca^2+^ spark frequency did not differ between WT and KI cells (*p* = 0.765, see Figures 3C,D), further pointing to the fact that activated Ca^2+^ handling does not require oxidized PKARIα. On the protein level, an unchanged phosphorylation status of Serine 2809 at the RyR2 was observed upon ATX-II exposure that was again not different between genotypes (Ser2809 is the PKA phosphorylation site at the RyR2, see Table 1 and Supplementary Figures S1A and SB). By contrast, increased CaMKII activity in terms of increased autophosphorylation at Threonine 287 was clearly present upon ATX-II, and accompanied by comparable CaMKII-dependent hyperphosphorylation of Serine 2814 at RyR2 in both genotypes (see Supplementary Material Figures S1 and SC–F).

**Table 1:** Expression and phosphorylation level of Ca^2+^ handling proteins.

	WT						KI					
	Vehicle	*n*	1 nmol/L ATX-II, 1 mmol/L CaCl_2_	*n*	1 nmol/L ATX-II, 2 mmol/L CaCl_2_	*n*	Vehicle	*n*	1 nmol/L ATX-II, 1 mmol/L CaCl_2_	*n*	1 nmol/L ATX-II, 2 mmol/L CaCl_2_	
pThr287 CaMKII/CaMKIIδ	1.000 ± 0.100	11	1.532 ± 0.190	13			0.919 ± 0.166	8	1.397 ± 0.147	11		
CaMKIIδ	1.000 ± 0.066	11	1.144 ± 0.126	13			1.255 ± 0.165	8	1.004 ± 0.060	11		
pSer2809 RyR2/RyR2	1.000 ± 0.083	7	1.007 ± 0.073	7			1.056 ± 0.027	6	1.120 ± 0.098	6		
pSer2814 RyR2/RyR2	1.000 ± 0.167	6	2.0130 ± 0.233	10			0.947 ± 0.079	6	1.636 ± 0.192	6		
RyR2	1.000 ± 0.055	7	1.098 ± 0.079	7			0.865 ± 0.084	6	1.009 ± 0.128	6		
pSer16 PLB/PLB	1.000 ± 0.067	7	1.051 ± 0.068	7			0.722 ± 0.074	6	0.895 ± 0.096	6		
PLB	1.000 ± 0.104	7	1.064 ± 0.121	7			1.086 ± 0.070	6	1.109 ± 0.084	6		
PKA RIα dimer/monomer	1.000 ± 0.117	7	1.207 ± 0.146	7	0.957 ± 0.079	7	n.d.	10	n.d.	10	n.d.	10

pThr287 CaMKII, CaMKII autophosphorylation site; CaMKIIδ, CaMKIIδ isoform; pSer2809 RyR2, PKA-dependent RyR2 phosphorylation site; pSer2814 RyR2, CaMKIIδ-dependent RyR2 phosphorylation site; RyR2, ryanodine receptor 2; pSer16 PLB, PKA-dependent PLB phosphorylation site; PLB, Phospholamban; PKA RIα, regulatory subunit of PKA; statistical analysis was performed using an unpaired *t*-test.

### ATX-II causes cellular arrhythmias in WT and KI cells to a similar extent

**Figure 4:**
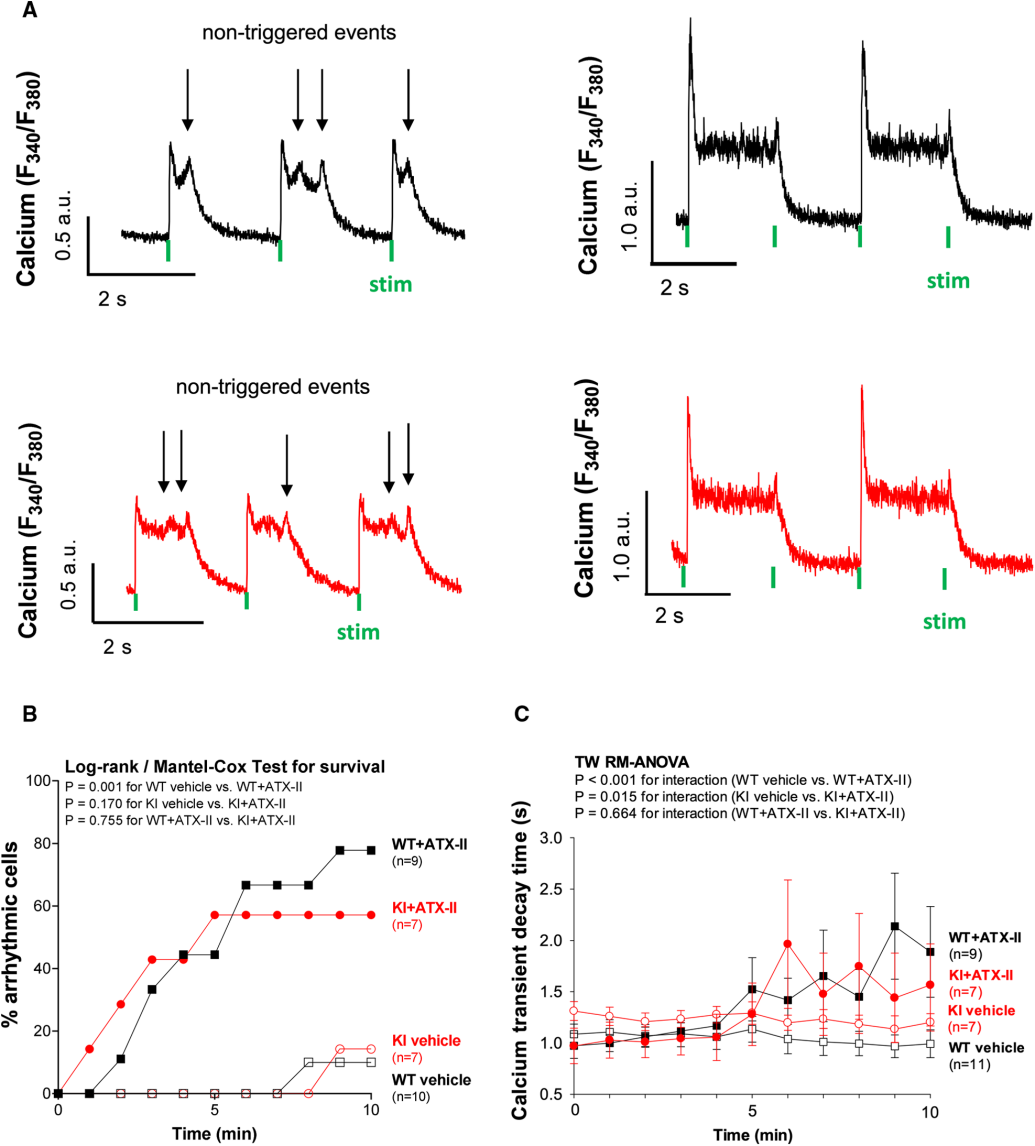
ATX-II causes cellular arrhythmias in WT and KI cells to a similar extent. (**A**) Original traces of non-triggered arrhythmic events during Ca^2+^ transient decay (left panel) and impaired relaxation (right panel) in ventricular myocytes of WT and KI mice loaded with Fura-2 AM upon treatment with ATX-II (1 nmol/L). (**B**) Percentage of ventricular myocytes with arrhythmias during exposure to ATX-II (1 nmol/L) for 10 min show a significant arrhythmogenic effect of ATX-II, which appears to be comparable in both WT and KI myocytes. (**C**) Decay time (RT90%, relaxation time until 90% decay of the Ca^2+^ transient) is significantly increased in ventricular myocytes of both WT and KI mice to a similar extent indicating impaired relaxation of cardiomyocytes upon treatment with ATX-II (1 nmol/L).

Since ATX-II is known to have proarrhythmogenic effects (26), we also analyzed cellular arrhythmias upon ATX-II treatment in our model [occurring as “non-triggered events” (24, 27, 28) as illustrated in Figure 4A, i.e., as spontaneous Ca^2+^ elevations during Ca^2+^ transient decay] to test whether oxidized PKARIα might exert a functional effect in that regard (see Figures 4A,B). As an overview parameter, we assessed the total proportion of arrhythmic cells in each minute at a certain point in time of the measurement (Figure 4B). By doing so, we observed that 78% of WT myocytes and 57% of KI myocytes developed cellular arrhythmias in terms of non-triggered events (as depicted in Figure 4A) within a time frame of 10 min of treatment with ATX-II, which, however, was not different between groups (*p* = 0.755 for interaction). Likewise, we did not observe a difference with respect to the ATX-II-dependently increased occurrence of non-stimulated Ca^2+^ waves between genotypes as assessed by confocal microscopy (*p* = 0.346, data not shown). This lack of change with respect to non-triggered arrhythmogenic events in case of absent oxidative PKARIα activation was mimicked by a similar delay in diastolic Ca^2+^ elimination that was comparably present in both genotypes upon ATX-II, but not different between WT and KI cells (*p* = 0.664 for interaction, see Figure 4C). In our *in vitro* model, PKA-specific phosphorylation of PLB at Serine 16 was not different upon acute ATX-II treatment as compared to the control and did not alter between genotypes (see Table 1).

### Unchanged I_Ca_ and absent PKARIα dimerization upon acute ATX-II treatment

**Figure 5:**
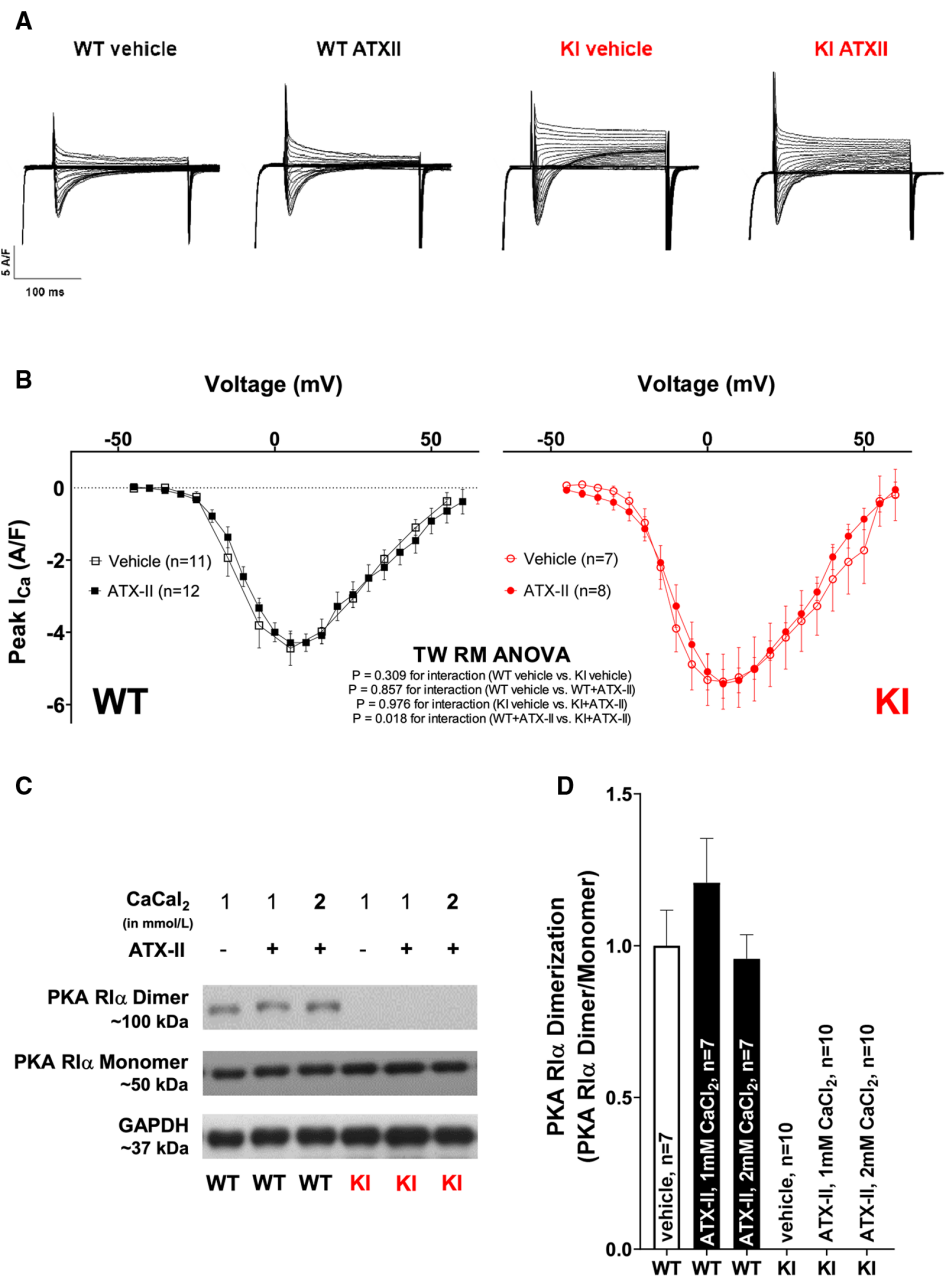
Unchanged I_Ca_ and absent PKARIα dimerization upon acute ATX-II treatment. (**A**) Original traces of I_Ca_ measured by whole-cell rupture-patch clamp technique in WT and KI ventricular myocytes upon treatment with ATX-II (1 nmol/L) vs. vehicle-treated control cells. (**B**) Mean data for peak I_Ca_-voltage relationship in isolated murine ventricular myocytes demonstrate unchanged I_Ca_ upon treatment with ATX-II (1 nmol/L) with no functional effect of absent PKARIα oxidation in KI cells. (**C**,**D)** Original Western blots and mean data for PKARIα dimer/monomer ratio show PKARIα dimer formation in WT murine ventricular myocytes upon vehicle treatment that is not significantly increased upon ATX-II treatment (1 nmol/L) upon 1 or 2 mmol/L CaCl_2_. Dimerization is completely absent in KI samples.

We have previously reported that oxidized PKARIα exerts a stimulating effect on the L-type-mediated Ca^2+^ current (I_Ca_) when acutely stimulated with angiotensin II (AngII) as well as in a long-term setting *in vivo* succeeding transverse aortic constriction (TAC) (23). Since I_Ca_ greatly contributes to the amplitude of the systolic Ca^2+^ transient, the relevance of I_Ca_ for the acute activation of Ca^2+^ handling upon ATX-II was investigated in a next step. As shown in Figures 5A,B and in Supplementary Material Figure S2, ATX-II did not alter the magnitude (Figure 5B) or the inactivation properties (Supplementary Material Figure S2) of I_Ca_ in WT myocytes. In contrast to our previous findings, we also failed to observe a functional effect on I_Ca_ in KI cells despite clearly increased ROS formation upon ATX-II (compare Figures 1C,D). Notably, we did not observe increased oxidation (i.e., dimerization) of PKARIα upon ATX-II in WT cells at 1 or 2 mmol/L Ca^2+^ (while no dimerization of PKARIα was observed in KI cells as expected, see Figures 5C,D). Hence, oxidation of PKARIα appears not to be required for the ATX-II-mediated acute activation of Ca^2+^ handling despite a clearly present pro-oxidant intracellular milieu.

## Discussion

In the present study, the role of oxidatively activated PKARIα in ATX-II-mediated Na^+^/Ca^2+^-mishandling in an *in vitro* model of acutely increased I_NaL_ was investigated for the first time. The main finding of our study is that oxidatively activated PKARIα does not contribute to the acute disturbance of Na^+^ and Ca^2+^ handling as induced by I_NaL_ (at least within a time frame of 15 min), which is accompanied by the unexpected fact that PKARIα does not become oxidized despite a clear pro-oxidant intracellular environment. We, therefore, conclude that oxidative activation of PKARIα is not crucially involved in mediating the positive inotropic and arrhythmogenic effects as induced by acute ATX-II exposure.

### Acutely increased I_NaL_ promotes enhanced oxidative stress

To induce an increase of I_NaL_, which typically occurs in heart failure, we treated mouse cardiomyocytes from WT and PKARIα KI mice with ATX-II. An increased I_NaL_ in heart failure is known to be associated with arrhythmias (29) and impaired Na ^+ ^-Ca^2+^ handling (4, 6). However, Na^+^ channels in ventricular myocytes are influenced by numerous factors. Among others, I_NaL_ is enhanced by CaMKII (30) and can be progressively induced by ROS (31, 32). Most importantly in regard to our study, protein kinase A is also involved in the regulation of Na^+^ channels (33, 34) and the modulation of I_NaL_ (35). Hence, PKA activation may potentially induce a positive feedback mechanism and further enhance I_NaL_. To exclude the possibility that treatment with equal concentrations of ATX-II affects ion currents in genotypes differently, we assessed the I_NaL_ in cardiomyocytes of both genotypes under control conditions and upon ATX-II treatment. Treatment with 1 nmol/L ATX-II resulted in an approximately threefold increase in I_NaL_ without significant differences between genotypes. Hence, it could be assumed that initial conditions were equal in both genotypes and that I_NaL_ is not subject to oxidatively-activated PKARIα (oxPKA)-dependent regulation upon acute ATX-II exposure.

We next tested whether ATX-II-dependently increased Na^+^ influx into the cell led to increased ROS formation in our setting as it was proposed previously (10). Various studies have shown that altered Na^+^ and Ca^2+^ handling in cardiomyocytes can lead to increased mitochondrial ROS production. Interestingly, there are several mechanisms leading to increased mitochondrial ROS production that may be relevant in our model, as follows: Increased cytosolic Na^+^ levels have been shown to reduce mitochondrial Ca^2+^ accumulation by promoting Ca^2+^ efflux through the mitochondrial Na^+^-Ca^2+^-exchanger (mNCE) (10, 11). Kohlhaas et al. demonstrated that decreased mitochondrial Ca^2+^ levels were associated with increased mitochondrial ROS production (10). In addition, it has also been suggested that enhanced oxidative phosphorylation during beta-adrenergic stimulation (that is needed to meet the increased energy demand) leads to increased electron leakage and thus mitochondrial ROS formation (36). In our present study, we found significantly increased mitochondrial as well as cytosolic ROS formation in both WT and KI myocytes following acute ATX-II exposure in the face of a comparable positive inotropic effect in both groups. We therefore interpret increased ROS formation in our model as the result of at least two mechanisms, namely, (i) cytosolic Na^+^/Ca^2+^ overload leading to mitochondrial ROS formation and (ii) enhanced oxidative phosphorylation in the mitochondria as a consequence of positive inotropy. Regardless of the exact mechanism, we clearly observed increased ROS production in WT and KI cells following ATX-II treatment so that we could largely exclude the possibility that ATX-II might have failed to increase ROS formation in one of the genotypes.

### The inotropic and arrhythmogenic effects of acutely increased I_NaL_ do not involve oxidation of PKARIα

We have previously found that oxidized PKARIα is an important factor for the maintenance of functional Ca^2+^ handling in the setting of enhanced oxidative stress by sustaining I_Ca_ (23). Hence, we now sought to determine the impact of oxidatively activated PKARIα on Ca^2+^ handling in the setting of acutely increased I_NaL_ with consecutively enhanced oxidative stress. In line with previous studies (9), we observed a positive inotropic effect upon ATX-II in terms of significantly increased Ca^2+^ transients and increased fractional shortening. In addition, we found an impeded diastolic decay of the Ca^2+^ transient that was paralleled by a time-dependent increase in arrhythmogenic non-triggered Ca^2+^ elevation events during Ca^2+^ transient decay, which altogether resulted in a delay of diastolic Ca^2+^ elimination. However, these effects were equally present in both genotypes, which speaks against a crucial involvement of oxidized PKARIα.

Likewise, ATX-II treatment induced diastolic SR Ca^2+^ leakage in both genotypes to a similar extent. An increase in SR Ca^2+^ leakage following enhanced I_NaL_ is generally in accordance with previous studies. Various factors such as activation of CaMKII (24) and direct ROS-dependent regulation of Ca^2+^ spark frequency (37) have been shown to modify this effect. Fischer et al. investigated the effect of I_NaL_ on the induction of SR Ca^2+^ leakage in atrial murine myocytes and uncovered that it was due to the activation of both CaMKII and PKA (38). By contrast, Wagner et al. ruled out a Nox2-dependent (and therefore redox-related) influence of PKA on SR Ca^2+^ leak and concluded that the leak was CaMKII-mediated (39), which is in accordance with another study from our group in which we have observed that acutely increased oxidative stress induces SR Ca^2+^ leakage independent of RIα dimer formation (23). Since we did not observe increased phosphorylation of Ser-2809 (which is the PKA-dependent phosphorylation site at the RyR2) here, it can be assumed that the increase in Ca^2+^ spark frequency upon ATX-II-related ROS generation as observed in our model is likely also not PKA dependent. Moreover, this finding indicates again that redox-activated PKA is not a major driver of SR Ca^2+^ leak in cardiac myocytes. Instead, in our experimental setting, ATX-II-activated CaMKII in WT and KI cells as well resulted in subsequently hyperphosphorylated RyR2 at the CaMKII-dependent phosphorylation site 2814 in both genotypes, which points to the fact that the induction of the ATX-II/ROS-mediated SR Ca^2+^ leak is primarily driven by increased CaMKII activity. In addition, ATX-II-dependent SR Ca^2+^ leakage may be a consequence of a sensitization of the RyR2 to the increased Ca^2+^ load of the SR (which increases the driving force for Ca^2+^ release for a given Ca^2+^ load of the SR that was also present as increased systolic fractional Ca^2+^ release).

Despite increased diastolic SR Ca^2+^ loss, however, SR Ca^2+^ content was still significantly elevated after treatment with ATX-II as compared with untreated control cardiomyocytes in both groups, which points to the fact that SR Ca^2+^ loading prevailed over SR Ca^2+^ loss in our model. Enhanced SR Ca^2+^ loading may be due to increased SERCA2a activity. In that regard, Eiringhaus et al. observed maintained, and upon CaMKII inhibition even increased, SR Ca^2+^ load despite I_NaL_-related diastolic SR Ca^2+^ loss, which they attributed to increased SERCA2a activity due to PKA-mediated PLB phosphorylation (9). However, in our model, we did not find a functional acceleration of SR reuptake [that can be approximated by Ca^2+^ transient decay kinetics in mouse myocytes (40)] nor evidence for PKA-dependent hyperphosphorylation of PLB (at Ser-16). This lack in ATX-II-dependent and PKA-mediated activation of central target structures of intracellular Ca^2+^ handling in our model may be explained by slightly different incubation strategies with ATX-II resulting in different exposure times to ATX-II (e.g., we did not add ATX-II to the fluorescent dye during incubation here). By contrast, we do not believe that this difference is due to different experimental Ca^2+^ concentrations, because we still failed to observe increased RIα dimerization following ATX-II even upon elevated Ca^2+^ concentrations as used by Eiringhaus et al. (compare Figures 5C,D). Conversely, since we observed clearly activated Ca^2+^ handling, we believe that PKA-dependent phosphorylation of PLB and RyR2 are at least not crucially involved in the very acute effects of ATX-II-dependent Ca^2+^ activation as observed here. Instead, in our murine model, diastolic Ca^2+^ overload and increased Ca^2+^ transient amplitudes are presumably a consequence of increased NCX activity in its reverse mode leading to severe cytosolic Ca^2+^ overload, since ATX-II does not enhance transsarcolemmal Ca^2+^ influx via the LTCC (which would be an alternative mechanism of cytosolic Ca^2+^ overload, see the following). While this effect may be different in larger animals and humans (41), our observation would be in line with previous reports that have demonstrated that inhibition of NCX significantly attenuates intracellular Ca^2+^ overload and also reduces arrhythmias upon increased I_NaL_ (30, 42). In contrast to our previous study (23), in which oxidized PKARIα maintained I_Ca_ upon oxidative stress, we failed to detect any functional effect of ATX-II treatment on the amplitude or the inactivation properties of I_Ca_ in both WT and KI cells here (while I_Ca_ was slightly but significantly enhanced in KI vs. WT upon ATX-II treatment in the face of a generally lower I_Ca_ amplitude in WT cells). Our observation that ATX-II-related ROS formation did not result in PKARIα oxidation in WT cells further suggests that this unexpected lack of functional effect on I_Ca_ is rather a result of absent oxidative PKARIα activation in comparison with a potentially differential regulation of I_Ca_ by oxidized PKARIα in the context of ATX-II.

### Pathophysiological implications and future perspectives

We acknowledge that our present study raises many new questions. Most importantly, it remains unclear why elevated cytosolic and mitochondrial ROS as acutely induced by ATX-II do not oxidize PKARIα, yet we believe that this aspect is beyond the scope of this study. Nevertheless, there is no question that this finding is in contrast to earlier studies in which an increased dimerization of the RIα subunit was clearly induced by various forms of oxidative stress such as by chronic pressure overload (23) or by pharmacologically induced oxidative stress using 1-nitrosocyclohexalycetate (NCA) (43). Interestingly, preliminary and as yet unpublished data by Simon et al. suggest that endogenous ROS indeed do not necessarily oxidize PKARIα in the heart, which appears to be correct for ROS as induced by NADPH oxidase 2 and 4 (NOX2 and NOX4), xanthine oxidase (XO), and the mitochondria (44). Instead, nitric oxide might turn out to be the relevant source for PKARIα dimer formation in the heart. Hence, our results may be at least hypothesis-generating in a way that it supports the notion that different stimuli and sources of ROS (or even NO) might differentially facilitate the dimerization of the PKARIα subunit. Conversely, we cannot rule out that chronically elevated I_NaL_ (such as in HF or in experimental models leading to HF such as TAC or myocardial infarction) would lead to PKARIα oxidation with consecutive functional consequences in intracellular Ca^2+^ handling and should therefore be subsequently investigated.

## Data Availability

The original contributions presented in the study are included in the article/Supplementary Material, further inquiries can be directed to the corresponding author.

## Supplementary material

Supplementary Figure S1 ATX-II activates CaMKII and leads to hyperphosphorylated RyR2 at the CaMKII-dependent phosphorylation site Serine 2814 in KI and WT cells (**A**,**B**) Original Western blots and mean data for PKA-dependent phosphorylation of Serine 2809 at RyR2 and for (**C**,**D**) CaMKII-dependent phosphorylation of Serine 2814 at RyR2 in the absence and presence of ATX-II. (**E**,**F**) depict increased CaMKII autophosphorylation at Threonine 287 (normalized to CaMKIIδ expression) following ATX-II exposure in WT and KI cells. * indicates significance between groups using unpaired Student’s *t*-test.

Supplementary Figure S2 Inactivation kinetics of I_Ca_ are unchanged upon acute ATX-II treatment. (**A**) Original traces of I_Ca_ inactivation at 0 mV as measured by whole-cell rupture-patch clamp technique in WT and KI ventricular myocytes upon treatment with ATX-II (1 nmol/L) vs. vehicle-treated control cells. (**B**,**C**) Voltage-dependent I_Ca_ inactivation kinetics displayed as mean values of τ_slow_ and τ_fast_ (double exponential fit) in isolated ventricular myocytes demonstrate no differences upon treatment with ATX-II (1 nmol/L) and no functional effect in case of absent oxidative PKARIα activation.
