# Supplementary figures and images for: The inotropic and arrhythmogenic effects of acutely increased late INa are associated with elevated ROS but not oxidation of PKARIα
Source: Front Cardiovasc Med. 2024 Jul 15;11:1379930. doi: 10.3389/fcvm.2024.1379930 (PMC11284163; doi:10.3389/fcvm.2024.1379930)

**A**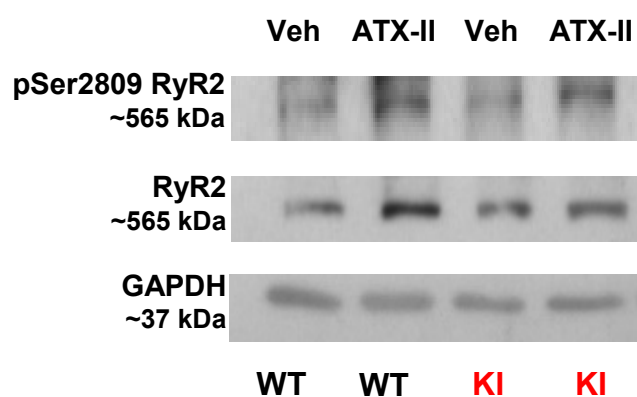**B**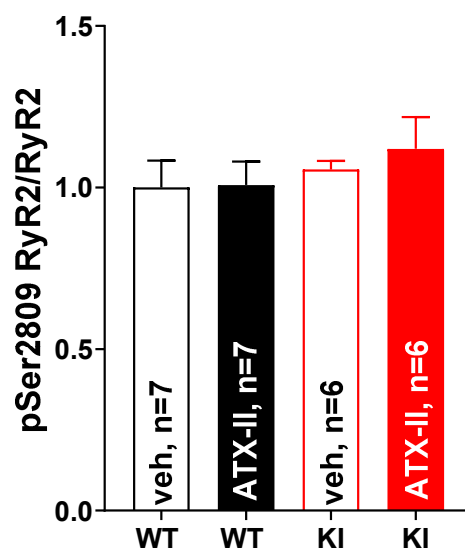**C**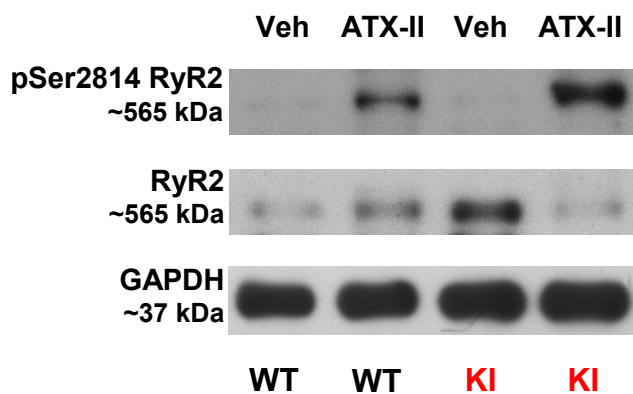**D**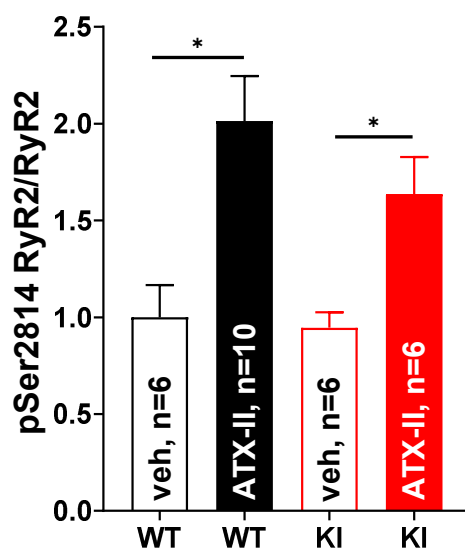**E**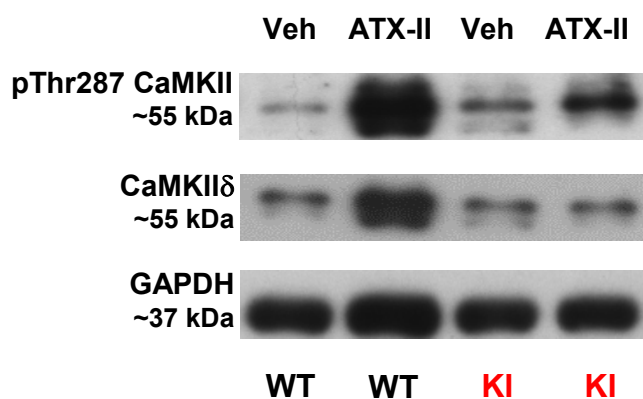**F**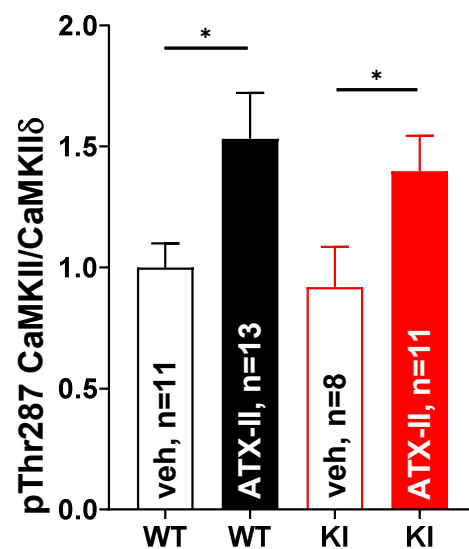**Supplemental Fig. S1**

Supplement: Supplementary Figure S1 — ATX-II activates CaMKII and leads to hyperphosphorylated RyR2 at the CaMKII-dependent phosphorylation site Serine 2814 in KI and WT cells (A,B) Original Western blots and mean data for PKA-dependent phosphorylation of Serine 2809 at RyR2 and for (C,D) CaMKII-dependent phosphorylation of Serine 2814 at RyR2 in the absence and presence of ATX-II. (E,F) depict increased CaMKII autophosphorylation at Threonine 287 (normalized to CaMKIIδ expression) following ATX-II exposure in WT and KI cells. * indicates significance between groups using unpaired Student’s t-test. [file Image1.pdf]

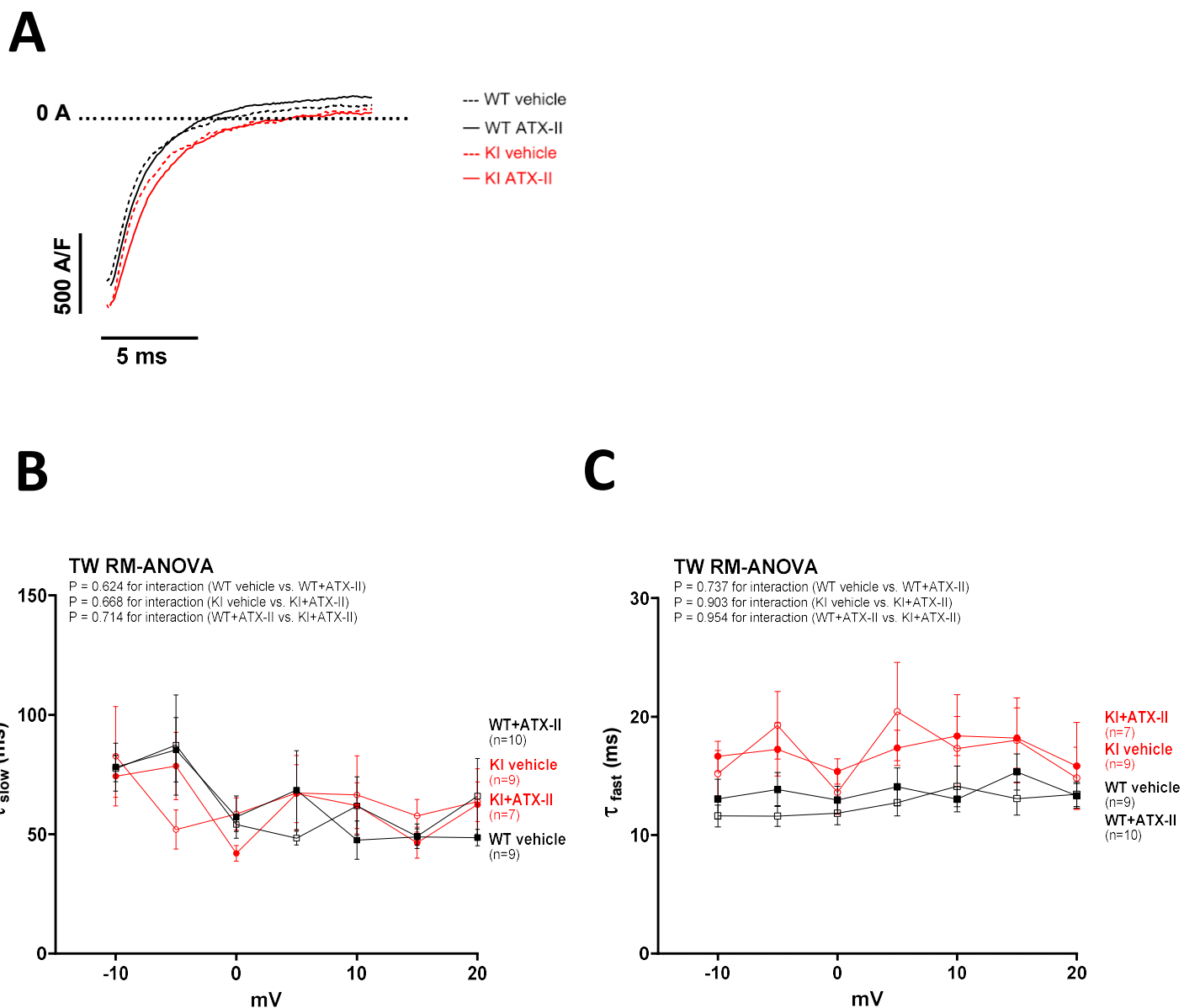

**Fig. S2**

Supplement: Supplementary Figure S2 — Inactivation kinetics of ICa are unchanged upon acute ATX-II treatment. (A) Original traces of ICa inactivation at 0 mV as measured by whole-cell rupture-patch clamp technique in WT and KI ventricular myocytes upon treatment with ATX-II (1 nmol/L) vs. vehicle-treated control cells. (B,C) Voltage-dependent ICa inactivation kinetics displayed as mean values of τslow and τfast (double exponential fit) in isolated ventricular myocytes demonstrate no differences upon treatment with ATX-II (1 nmol/L) and no functional effect in case of absent oxidative PKARIα activation. [file Image2.pdf]
